# Reviews of Books

**Published:** 1924-01

**Authors:** 


### REVIEWS OF BOOKS.

### A CHINESE ODYSSEY.

**The Travels of Fa-hsien or Record of the Buddhist Kingdoms.** Retranslated by Professor H. A. Giles, M.A. (Camb. University Press. 5s. net.)

From the earliest days mankind has felt a *Wanderlust* which has compelled it to wander over the face of the earth. Whether this compulsion has shown itself as a desire to seek "fresh woods and pastures new," or as a search for knowledge of new lands and strange folk, or as a mere love of personal gain, unquiet spirits have ever ventured far afield. But in these modern days the earth's circumference has contracted, and the difficulties and dangers of travel have become so attenuated that we, who light heartedly take long journeys from one side of the world to the other, can scarcely envisage the courage of early travellers.

There is no part of the earth the exploration of which begets enthusiasm more than any other than that great area of Central Asia north of the Himalaya Range. It is still, despite the journeys of Sven Hedin and Sir Aurel Stein, practically unknown. Yet it was by the great trade route through these lands that the East and West were linked together. The famous Chinese pilgrim, Hsüan-tsang, in the seventh century, and the Venetian traveller, Marco Polo, in the thirteenth, have left us chronicles of their travels. But Chinese records have preserved for us the story of a still earlier traveller. A pious Buddhist monk, Fa-hsien, distressed by the imperfect familiarity of his compatriots with the Buddhist Scriptures, undertook a journey with a small body of similarly minded enthusiasts which drew him from Central China, across the desert of Gobi, by Khotan and Kashgar, over the Hindu-Kush through India to the mouth of the Hoogli, and thence by ship to Ceylon and Java, and finally to Canton. His journey, the land part of which was almost entirely on foot, lasted from 399 to 414 A.D., but he accomplished the object of his journey by returning with what he had set out to secure, copies of the Buddhist canon and images of the Buddhist deities.

Dr. H. A. Giles, Professor of Chinese at Cambridge, has published a re-translation of Fa-hsien's own account of his religious pilgrimage. On his way he visited all the principal places rendered sacred by their traditional association with the earthly life of Buddha, and of these he writes with all the enthusiasm of the true disciple. He piously relates the legends and miracles which have been told him, and mentions all the monasteries, in many of which he had been entertained. In his simple story there appear from time to time little human touches which betray his longing for his own home in China. Thus concerning his stay in Ceylon he writes :—

> Fa-hsien had now been many years away from his own land of Han; the people he had to deal with were all inhabitants of strange countries; the mountains, the streams, plants and trees on which his eyes had lighted were not those of old days; moreover, those who had travelled with him were separated from him—some having remained behind in those countries, others having died. Now, beholding only his own shadow, he was constantly sad at heart; and when suddenly, by the side of this jade image he saw a merchant make offering of a white silk fan from China, his feelings overcame him and his eyes filled with tears.

In the long voyage home Fa-hsien had many hair-breadth escapes, and was barely saved from the fate of Jonah of being cast ashore. But the perilous voyage came to an end at length, when the ship made the promontory of Shantung in the neighbourhood of Kiao-chow. "And now, after having passed through much danger, difficulty, sorrow and fear, suddenly reaching this shore and seeing the old familiar vegetables, they knew it was their fatherland." Fa-hsien ends his story on a note of thankfulness for the Divine protection manifested to him in his preservation in the hour of danger. At the instigation of a friend he was persuaded to commit his story to writing: "Therefore he wrote down on bamboo tablets and silk an account of what he had been through, desiring that the gentle reader should share this information."

Poor Fa-hsien was unfortunate in his name. A later Chinese commentator writes of him under the name Fa-ming. The Descriptive Catalogue of the Imperial Library (published in 1795) gives the reason for this change of name. "He did so because the character *hsien* had been appropriated by the Emperor Chung Tsung (its use was therefore taboo for other persons) and men of the T'ang dynasty had substituted *ming*. For this reason there occur in the original commentary the four words, changed because Imperially appropriated,"—surely this is one of the strangest vicissitudes which ever befel an author!

Professor Giles has added an introduction. This is, however, too short and cursory to be of much value to the general reader. A few explanatory notes on Buddhism and on sundry expressions such as going into "summer retreat" and the doctrine of the three vehicles would have had more interest than his dissertation on the Christian doctrine of the Trinity which appears scarcely germane to the subject of the book. Fa-hsien's story is fascinating in its naive simplicity and directness.

### DISTURBED REFLEXES.

**Angina Pectoris.** By Sir James Mackenzie, F.R.S., F.R.C.P. (Oxford Medical Publications. 30s. net.)

The principles governing the methods of research undertaken in the St. Andrew's Institute for Clinical Research, of which the distinguished author of the present volume is the Director, comprise, among others, a comprehensive study of the mechanism of pain and its relation to disturbance of the function of a given organ, more especially in regard to prognosis.

Working upon these lines, Sir James Mackenzie has built up, on the sure foundation of clinical evidence, the theory of disturbed reflexes as a cause of disease, and in this illuminating monograph he shows the significance of certain associated phenomena in the study of angina pectoris. A state of hypersensitiveness of the nervous system is a common condition in angina, and this is prone to manifest itself in hyperalgesia of various parts of the body. In one case recorded by the author the typical attacks were produced by turning in bed or by taking part in conversation. The existence of " centres," or limited regions capable of producing a complicated series of responses, is demonstrated, and these may become so sensitive as to evoke such responses as vomiting, asthma, or angina pectoris. The mechanism of this disorder is inherent in every individual, but the characteristic symptoms which constitute a morbid entity only appear when the normal activities are thrown out of gear. Overstrain, progressive changes in the vessels, and toxic infections are among the factors that tend to produce the peculiar contraction of the heart muscle that is the fundamental agent giving rise to the fully developed angina. Sir James refers to John Hunter's well-known affliction, and gives him as an illustration of the power of mental excitement in causing an attack, which in his case proved fatal.

In a series of cases investigated by the author the majority died within five years from the onset of illness, the average age at death being between 61 and 65. Primary angina is relatively rare in women, while secondary angina is frequent. Seeing that the condition is due to organic changes in the heart that are irremediable, the prognosis is largely based upon the degree of response to effort, but it should be borne in mind that symptoms may not appear until the disease has advanced to such an extent as seriously to embarrass the work of the heart. Not the least valuable part of the book is the detailed analysis of 160 case-records, many of which are accompanied by cardiograms and pulse-tracings. Altogether this is a book to read again and again.

### POISONS AND POISONERS.

**Poison Mysteries in History, Romance and Crime.** By C. J. S. Thompson, M.B.E. Illustrated. (The Scientific Press. 10s. 6d. net.)

Mr. Thompson, who has already published an attractive volume on the romance of alchemy and pharmacy, long ago found a cognate subject in poisons and poisoners, and has here treated it afresh in sprightly and entertaining fashion. At first sight it would seem strange indeed that romance should cling to any form of murder, but into cases of poisoning the element of mystery and uncertainty frequently enters, and human nature dearly loves a mystery. In days when little was known of chemistry and the first toxicologist was as yet unborn, the man or woman who knew something about poisons and their properties possessed a weapon of enormous value for which Kings and Princes, and even Popes, were very ready to pay. People might be suspicious when a great personage died suddenly or mysteriously, but methods of detecting poison in the body were crude and empirical, and the poisoner and his patron usually escaped. On the other hand, poison was often suspected where it had no existence, as in the case of Henrietta, Duchess of Orléans, the sister whom Charles II. so dearly loved, and over whom Bossuet pronounced one of his most splendid orations. Her death was attributed to diamond dust mixed with sugar and placed upon strawberries, but diamond dust is not a poison, and there never was any serious evidence that this charming woman, from whom most living royal personages descend, died from other than natural causes.

Italy, in what are loosely called the Middle Ages, was the classic land of the poisoner, and France took this, along with many other fashions, from her. If Italy had the Borgias, France had the Marquise de Brinvilliers and La Voisin. The amazing brother and sister, Cesare and Lucrezia Borgia, children of Pope Alexander VI., are, perhaps, the most famous poisoners in history, though it is certain that many crimes were attributed to them of which they were innocent—Lucrezia, especially, seems to have been much less black than she has been painted. Between poisoning for reasons of State and poisoning as a profession there is no moral distinction, and there never was so professional a poisoner as the woman Toffana, whose "Aqua Toffana" is reported to have " removed " hundreds of people in Naples and elsewhere. Its active constituent was arsenic and, being freely sold as a cosmetic, those who were afflicted with troublesome relations or unfaithful lovers " always had something to quiet them handy." For a century or two there was in Italy a mania for poisoning, and that strange Battista Porta, who was always hovering on the fringe of the occult, in the book he wrote on the subject in 1589, describes some of the favourite poisons of his day :—

> He mentions various means for drugging wine, a favourite medium for administering poison. For this purpose belladonna root, nux vomica, aconite and hellebore were employed, all of which are very deadly in their effects. He gives a formula for compounding what he calls a very strong poison named " Venenum Lupinum," which was composed of aconite, taxus baccata, caustic lime, arsenic, bitter almonds and powdered glass. These substances were to be mixed with honey into a stiff paste and made into pills the size of a hazel nut.

The sixteenth century pill must have been prodigious indeed if one the size of a hazel nut failed to awake suspicion. Living people who never heard of Italy or its poison record know all about the uses of honey in poisoning. Take the Malays, for instance :—

> The natives are accustomed to use poison in the same manner as employed in ancient times—namely, by mixing it with honey which is sometimes smeared on the under surface of a knife. The poisoner then shares a meal with his enemy and divides a water melon in half with the poisoned blade, but is careful to eat only the upper and harmless portion as his share of the fruit.

But methods of poisoning are endless. Gloves and rings, flowers and candles, have all been used in fact or in fiction, and Mr. Thompson gives some entertaining extracts from such famous stories as *Monte Cristo* and *The Woman in White.* The inimitable Count Fosco, himself a poisoner of considerable accomplishment, declares that " chemists might sway, if they pleased, the destinies of humanity "—so long, of course, as there are not other chemists hot on their track. Chemistry has " got even " with most poisons, though it is not unlikely that new ones will be evolved, in their turn to be discovered. Mr. Thompson, indeed, tells us of one :—

> The most deadly poison known to science to-day exists in the form of an innocent-looking white powder which is highly dangerous even to handle. It emits a slight vapour even when exposed to the air, which if inhaled would cause instant death. It has been estimated that if three grains were diffused in a roomful of people it would kill every one present. It is hardly necessary to state that poisons of such virulence as those revealed by modern chemical research were unknown to the chemists of the Middle Ages, and it is equally certain that the latter knew of few poisonous bodies that are not familiar to the chemists of to-day.

In the wide field of poisoning Mr. Thompson has left little unexplored, and his book is full of interesting and often thrilling stories of the distant past and of a past which is but that of yesterday. He devotes many chapters to the famous poisoning cases of the last half century, some of which we should have liked him to tell in somewhat greater detail. How near he comes to our own hour is indicated by what he has to say of poison gas and poisoned sweetmeats dropped from aircraft in the late war, while he winds up with the poison aspects of the very recent Ilford case. His book is full of curious and unfamiliar lore, and it is dedicated, appropriately enough, to that ruthless tracker down of poisoners, Sir William Willcox.

### THE MOST PRIMITIVE SENSE.

**Aromatics and the Soul.** By Dan Mackenzie, M.D. (Heinemann. 7s. 6d.)

This little treatise concerns one of the elementary subjects of which little is known. Though the sense of smell is perhaps the most primitive possessed by sentient beings, its organ remains apparently unchanged from the earliest type. Besides this, the olfactory organ is better developed in the simpler forms of animal life, and human beings to this day possess very few words to describe the different kinds of scents or smells that they perceive. There is no vocabulary and no art of olfaction, though the senses of sight and hearing possess both. Certain insects and animals, however, depend much upon it for their knowledge of the world. In man it is not only simple, but mysterious, if only for its strange power of evoking memory. To Dr. Mackenzie's quotations from the poets in illustration of this, we may add one from D. G. Rossetti :—

I have been here before,
But when or how I cannot tell :
I know the grass before the door,
The sweet, keen smell,
The sighing sound, the lights around the shore.

In his chapter on "Smell in Folk-lore, Religion and History," Dr. Mackenzie discusses the odour of sanctity, and states that "a piny aromatic odour, of considerable strength, is occasionally noticeable in certain people, and I can myself testify that it becomes stronger on the approach of their death." He believes that dogs wallow in ordure for the same reason that ladies place orris-root in their baths : to give pleasure to their neighbours. Dr. Mackenzie suggests that certain well-known phenomena of crowd psychology, whether the crowd be one of worshippers or an angry mob, may be caused by obscure and mutual stimulation of the olfactory sense of their members. He discusses different theories of olfaction and the attempt made to classify odours. It is all interesting, and forms a good introduction to the subject, because of the numerous references in the book to other writers. The subject in all its ramifications is evidently a hobby with Dr. Mackenzie, and he has added to his acute personal observations much learning, so that anyone who is drawn to these pages will keep the book for reference and for the sake of the curious information that it contains.

### A HOSPITAL HISTORY.

**A Short History of the Royal Southern Hospital, Liverpool.** By C. J. Macalister, M.D., F.R.C.P. (W. B. Jones and Co., Liverpool.)

The senior physician of the Royal Southern Hospital, Liverpool, has written a history of the institution to commemorate the jubilee year of its existence in the present building. Originally founded in 1838, it was opened in 1842 to serve the growing population round the docks at the South end of the city. A ward still commemorates the concert at which Jenny Lind sang on the hospital's behalf in the early days of its history. By 1857 it attained 100 beds, and gas was then first used to light the wards. Three years later self-acting sanitation was introduced. In 1872 a new hospital was opened, and its modern career begun. Seven years later Dr. Macalister became a student there, and his history is much enlivened by the personal recollections which he weaves into its story. Among his recollections is the strange fact that until 1890 the operating theatre was placed immediately over the mortuary and post-mortem room.

The most interesting pages are those concerning the hospital's physicians and surgeons of the past, for their experiences bring before the reader the conduct of the institution more vividly than could a bare record of extensions and official dates. Indeed, a wide acquaintance with institutional histories leads us to believe that much is to be said for entrusting to members of the medical staff the writing of hospital histories generally. Dr. William Carter, who joined the staff in 1870, discovered the poisoning of Mr. Maybrick by arsenic in 1889, and there are many stories of equal interest which ensure that this history will be found acceptable reading by anyone into whose hands it may come.

### THE ADMINISTRATION OF ANÆSTHETICS.

**Practical Anæsthetics.** By H. Edmund G. Boyle, O.B.E., M.R.C.S. (Eng.), L.R.C.P. (Lond.), and C. Langton Hewer, M.B., B.S. (Lond.), M.R.C.S. (Eng.), L.R.C.P. (Lond.). (Henry Frowde and Hodder and Stoughton. 3rd Edition. Price 6s. 6d. net.)

The third edition of this extremely practical little handbook has been considerably increased in value by having been almost completely rewritten, and by the collaboration of Dr. C. Langton-Hewer with its original author. Though of late years the only fresh anæsthetic agent which has been used at all universally is ethyl chloride, in no branch of medicine perhaps has so great an advance been made and so much new work been done in devising fresh methods of administering the drugs and in improving the old ones. The chapter on Blood Pressure during Anæsthesia, specially written for this edition by Dr. E. I. McKesson, of Toledo, Ohio, an expert on the subject, will be read with interest. By the graphic record supplied by the sphygmamometer throughout an operation the onset of the danger-point in circulatory depression can be accurately ascertained, and intravenous administration of saline solution started in time to prevent the worst consequences of shock. The blood pressure alone also determines the proper quantity of solution to be used.

The book should be useful not only to the anæsthetist, but to the general practitioner, the house-surgeon and the nurse. A copy might well find its way into the anæsthetic-room, so that the theatre-sister, on whom so much of the successful conduct of an operation depends, should have the opportunity of becoming thoroughly familiar with the most up-to-date methods of giving general and local anæsthetics.

### A DOCTOR'S GUIDE TO THE LAYMAN.

**Good Health and Long Life.** By Cecil Webb-Johnson, M.B. (Methuen. 3s. 6d.)

Mr. Cecil Webb-Johnson's book is an essay in scientific commonsense, and, on the whole, he is as sensible in his remarks on dress, tobacco and the digestive and appetising value of sound wine and light beer, as he is on sleep, diet, or constipation. For science, no less than nescience, has its faddists, and the author is valuable who successfully avoids the opposite errors of the two. Against Dr. Johnson's "shameless" advocacy of tea, Dr. Webb-Johnson sets Cowper's no less exaggerated warning, and is scientifically content to caution us against excess without pretending that the abuse of a food means that the food is bad! A wide general culture is revealed in these pages, and to this is due the balance of the argument throughout. To understand medicine one needs to understand everything else, and the simplicity and aptness of illustration, coupled with an incisive style, make this the soundest (and most amusing) guide to the layman that we have seen for some time. In it resides a reflective mind, nourished on a wide clinical experience.

### MISCELLANEOUS NOTICES.

### A Directory of Tuberculosis.

**Handbook of Tuberculosis Schemes for Great Britain and Ireland. (National Association for the Prevention of Tuberculosis, 20, Hanover Square, W. 1. 10s. 6d., post free.).—It is sufficient to say of this book that it is well arranged, clearly and spaciously printed, and not overloaded with extraneous matter. It is an indispensable work of reference for all authorities engaged in tuberculosis administration.**

### A Medical Annual.

The Medical Year Book for 1924. Edited by Charles R. Hewitt. (Heinemann. 12s. 6d. net.)—A classified directory of this kind may be trusted to save its owner a great deal of time, and though not an official publication the information it contains is thoroughly dependable. Its lists of specialists and consultants, of universities, hospitals, medical schools and post-graduate institutions are most valuable, and there is indeed no side of medical work which is left untouched. It is an annual without which a library of reference books is incomplete.

### Some Fiction.

Heristal's Wife. By Cecil Adair. A Young Autocrat. By Cecil Adair. The Heroine of Chelton School. By May Wynne. (Stanley Paul. 7s. 6d., 2s. and 2s. respectively.).—Cecil Adair is known apparently as the "Joy of Life" novelist, but it is not life which she describes, but some blameless if rather enervating existence in which everyone is very good, very sentimental and excessively garrulous. She is less exuberant when dealing with children, and *A Young Autocrat* is a pleasant little tale enough. Miss Wynne's story of school life is quite excellent, and both the cheaper books are nicely bound and printed for their very moderate price.

### A Nursing Encyclopædia.

The Nursing Mirror Pocket Encyclopædia and Diary. (The Scientific Press, Ltd., 28-29 Southampton Street, Strand, W.C. 2. 1s. 8d. post free.)—Each year the encyclopædic part of this useful little book grows larger as fresh items of interest are added to it, so that in the Diary for 1924 we find several additional pages of reading matter, bringing the total number up to 338. Among the newest items we note the latest information on State Registration matters and excellent reproductions of the badges issued to registered nurses by the General Nursing Council for England and for Scotland. The College of Nursing Badge is also reproduced with particulars as to the correct manner of wearing it. There are details concerning the proper equipment for the bags of visiting nurses and of midwives; and the right way to treat the common and troublesome complaint of chilblains besides others too numerous for inclusion in a short review.

### Septic Teeth.

The Diseases of the Periodental Tissues Due to Infection in their Relation to Toxæmia. A series of lectures delivered under the auspices of and published by the Dental Board of the United Kingdom, 44, Hallam Street, W. 1.—These four lectures, which were previously published in various journals, are here collected and the result is a highly interesting work. The Patho-histology is first dealt with by Dr. J. Howard Mummery, the local clinical symptoms are reviewed by Mr. J. G. Turner, of the Royal Dental Hospital, Sir Wm. Willcox discusses the systemic effects, and Professor E. E. Glynn gives a most valuable description of the various causal organisms and our present knowledge concerning the part they play in dental disease. In view of the clinical importance usually attached to the absorption of "toxins" from diseased and septic teeth this combined attack upon and detailed exposition of the subject serves an immeasurably useful purpose. We can assure the physician and the pathologist that these lectures are of the first order of excellence.

### An Appeal to the Public.

Homœopathy: What It Means to You and Your Health. By C. Fraser McKenzie, C.I.E. (The Homœopathic Publishing Company.).—This small 96-page book is intended for lay readers and is dedicated to "All Broad Minded Medical Students in whose Hands Lies the Future of Our Medical System." The book is an appeal to the public over the heads of the medical profession, or at least that section of it which does not conform to homœopathy. The author evidently is somewhat of a dramatist, and he adds colour to an already vivid and interesting subject by introducing his readers to villains, heroes and defenceless persons towards whom one may act villainously or heroically according to temperament. The chief villains of the piece are, of course, the members of the General Medical Council and allopaths of various colours. The rescue of the innocent public is left to the homœopathic practitioner whose brilliant successes in the treatment of cholera and other diseases have in the past, according to the author, been deliberately suppressed. In this connection it is said of the Medical Council that: "They actually concealed the cholera statistics of the Homœopathic Hospital, but a vote of Parliament forced them to produce these." The author insists that, though homœopathic principles have been the subject of ridicule and invective, he has searched in vain for a logical and reasoned contradiction. The author's own logical and reasoned advocacy of homœopathy is often plausible, and it will have served a useful purpose if it forces the conventional allopath to study the claims of homœopathy in the light of recent research, most of which, it must be admitted, has been carried out by the much abused allopath.

